# Regulation of Actin Dynamics in the *C. elegans* Somatic Gonad

**DOI:** 10.3390/jdb7010006

**Published:** 2019-03-20

**Authors:** Charlotte A. Kelley, Erin J Cram

**Affiliations:** Department of Biology, Northeastern University, Boston, MA 02115, USA; kelley.ch@husky.neu.edu

**Keywords:** actin cytoskeleton, *C. elegans*, spermatheca, sheath, contractility

## Abstract

The reproductive system of the hermaphroditic nematode *C. elegans* consists of a series of contractile cell types—including the gonadal sheath cells, the spermathecal cells and the spermatheca–uterine valve—that contract in a coordinated manner to regulate oocyte entry and exit of the fertilized embryo into the uterus. Contraction is driven by acto-myosin contraction and relies on the development and maintenance of specialized acto-myosin networks in each cell type. Study of this system has revealed insights into the regulation of acto-myosin network assembly and contractility in vivo.

## 1. Introduction

The actin cytoskeleton is a dynamic structure polymerized from globular actin monomers (G-actin) into a helical filament (F-actin) [1]. The inherent polymerization and depolymerization characteristics of actin and a suite of actin-interacting proteins—including nucleation, elongation, capping, and disassembly factors—enable rapid remodeling of actin-based structures [2]. Actin filaments play important roles in cell division and cytokinesis, [3,4,5], and are essential components of many cellular structures including cortical actin networks, contractile acto-myosin stress fibers, lamellipodia and filopodia [6,7,8,9,10]. These structures play an important role in cell migration [11], tissue morphogenesis [12,13,14], wound closure [15], and cell and tissue adaptation to physical stress [8,16].

Both cell culture [17,18,19] and in vivo [20,21,22] studies have shown that the actin cytoskeleton reorganizes in response to cues from the mechanical microenvironment. Stress fibers, contractile acto-myosin bundles, are common in cells exposed to physical stress [9] and have been well-studied in the context of cell migration [8,23,24,25]. However, much less is known about the regulation of contractile stress fiber-like structures in non-migratory cells [23,26], and few in vivo systems have been developed to allow real-time investigation of actin dynamics in intact contractile tissues [21,22,27,28].

Many insights into actin regulation in vivo come from elegant studies of cell movements and cell contractility during Drosophila elongation, gastrulation, egg chamber rotation, and tracheal tube development [14,20,21,22,27,28]. Studies in *Xenopus* embryos have shown the importance and regulation of actin dynamics during cytokinesis and furrow formation [5,29], in maintaining tight junction barrier function during cell elongation and shape change [29,30], and in wound repair [31,32,33]. Although cell shape changes and large perturbations to the localization of the acto-myosin contractile apparatus can be observed in these cell types, the small size of these cells makes imaging of dynamic reorganization of the actin cytoskeleton a challenge.

The nematode *C. elegans* was established as a model organism for developmental genetics and neurobiology because of its rapid life cycle, generous production of progeny, and ease of propagation in the laboratory. Since then the almost invariant cell lineage, optical transparency, ease of transgenesis, and availability of cell-specific fluorescent markers have made ‘the worm’ an amenable model for cell biology, particularly, for the analysis of individual cells in a living animal. Excellent recent reviews highlight the role of the actin cytoskeleton in *C. elegans* cell migration [34], neurodevelopment [35], and the striated muscle of the body wall [36]. Here we focus on the actin structures found in the somatic gonad of the *C. elegans* hermaphrodite.

**Figure 1:**
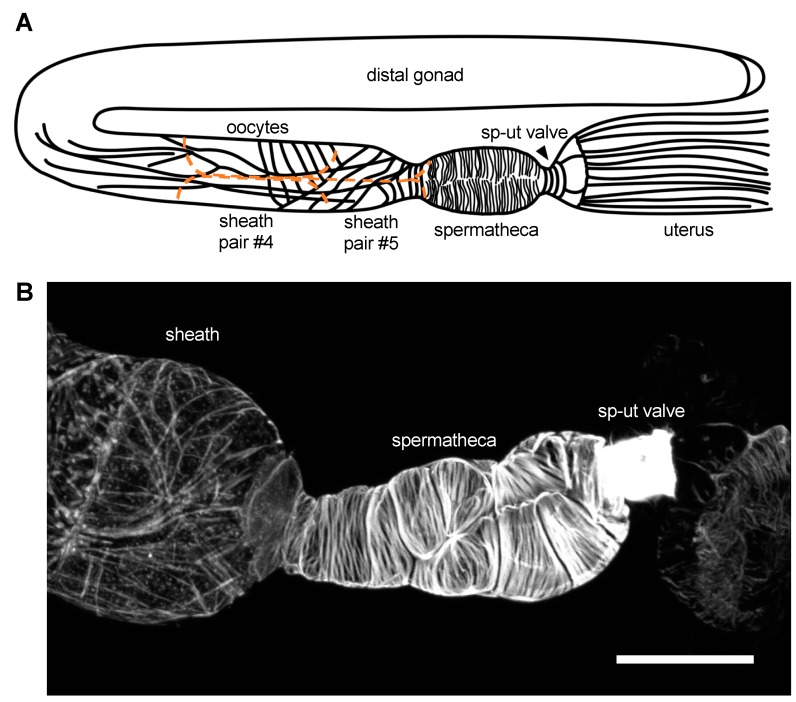
Actin networks in the *C. elegans* somatic gonad. (**A**). Diagrammatic representation of the *C. elegans* gonadal anatomy. Sheath cells are outlined in orange and oocytes are in grey. (**B**). Phalloidin stain of a dissected *C. elegans* gonad, demonstrating the actin structures observed in the sheath, spermatheca, and spermathecal–uterine (sp–ut) valve. Scale bar 20 micrometers.

The somatic gonad in the *C. elegans* hermaphrodite is a bilobed structure surrounded by a single layer of contractile cells [37]. Each gonad ‘arm’ is composed of a distal tip cell (DTC) which caps the distal end of the structure and forms the mitotic germ cell niche; gonadal sheath cells which surround the developing oocytes; the spermatheca, which stores sperm and is the site of fertilization; and a spermathecal–uterine (sp–ut) valve, which connects each gonad arm to a common uterus (Figure 1). The sheath, spermatheca, sp–ut, and uterus are connected by gap junctions [38,39] and coordinate to propel fertilized embryos through the system. Although contractility in all cells of the somatic gonad is driven by myosin, the regulation of contractility and the structural arrangement of the acto-myosin bundles in each cell type is strikingly different (Figure 1). Because the cells of the gonad are large and clearly visible in the intact animal, study of this system has revealed key insights into the question of how actin and myosin are assembled into the radically different networks that regulate tissue function. In addition, because the gonad naturally stretches and contracts during the ovulation process, this system is ideal for studying how cells in intact tissue differentially regulate their acto-myosin cytoskeletons in response to stretch and contraction.

## 2. The Gonadal Sheath Cells

Five pairs of thin sheath cells form a single layer of cells, which migrate out and enclose the germ line during the larval stages L3 and L4 [40,41,42]. In addition to providing structure to the gonad, the sheath cells promote gametogenesis and germ line proliferation [41,43,44,45]. Each pair of sheath cells has different properties; here we focus on the most proximal three pairs of sheath cells, which enclose the maturing oocytes and drive ovulation. The actin in sheath cell pairs #3–5 is predominantly longitudinally aligned, with a network of long, thin, interconnected filaments. In addition, pair #5 has circumferentially aligned actin bundles at the interface with the spermathecal neck (Figure 1) [41,46].

Contraction of the sheath cells drives the oocyte into the spermatheca, in what appears to be a push-pull effort where sheath cells #3–4 contract and push the oocyte forward while the most proximal sheath cells (#5) pull the spermathecal neck over the entering oocyte [41]. The actin arrangement in these cells enables these contractile events. Even though the actin in the sheath cells is non-striated, and morphologically similar to acto-myosin networks in smooth muscle [46], contraction of the sheath cells is driven by a mechanism that shares features of both smooth muscle and striated muscle. Current models, developed from in vivo imaging data and analysis of genetic interactions, suggest that both calcium signaling and activation of the small GTPase RHO-1/Rho are required. Binding of the epidermal growth factor LIN-3 to LET-23, a tyrosine kinase receptor, induces sheath cell contractions by activating the phospholipase PLC-3/PLCδ [47,48]. Cleavage of the phospholipid phosphatidyl inositol 4,5-bisphosphate PIP_2_ by PLC-3 produces inositol triphosphate (IP_3_), which leads to activation of the ITR-1/IP_3_ inositol triphosphate receptor and the release of calcium from the endoplasmic reticulum [47]. Similar to smooth muscle, RHO-1/Rho activation of LET-502/Rho kinase (ROCK) leads to the phosphorylation of the MLC-4/myosin regulatory light chain and activation NMY-1/non-muscle myosin II (NM II). Rho activity is regulated by the guanine nucleotide exchange factor GEF, VAV-1 [49]. Similar to striated muscle, sheath cells #3–5 also express NMY-2/myosin, the UNC-54/MYH3 and MYO-3/MYH3 myosin heavy chains [50,51,52] as well as LEV-11/tropomyosin and the troponin complex (PAT-10, UNC-27, TNI-1, MUP-2) typically found in striated muscle [51,52,53,54]. In the sheath, calcium signaling activates myosin contraction both by stimulating phosphorylation of the regulatory light chains, and through the relief of steric hindrance by the troponin complex [54,55].

Sheath cells contain focal-adhesion like foci known as dense bodies [51]. Focal adhesions are integrin-based, structural and signaling multi-protein complexes that connect the actin cytoskeleton to the extracellular matrix [56]. In adherent mammalian cells, acto-myosin bundles called stress fibers terminate in focal adhesions [56,57]. In the sheath, the actin bundles seem to intersect, but not terminate in, sheath cell dense bodies [51]. PAT-3/β integrin and DEB-1/vinculin, both core focal adhesion proteins, co-localize in puncta on the actin filaments [51]. The matrix component UNC-52/perlecan is also localized to these structures [51]. RNA interference (RNAi)of these proteins results in decreased contractility and oocyte entry defects [51] and efficient depletion of PAT-3/β integrin and PAT-4/integrin linked kinase leads to a clear loss of F-actin in the sheath [58]. The sheath also expresses TLN-1/talin, an adaptor protein that helps to connect integrins to the actin cytoskeleton. Although it is not known if TLN-1 localizes to dense bodies, the circumferential actin bundles in the sheath cells are completely disrupted when TLN-1 is disrupted by RNAi [59]. Given the significant conservation of these components, it seems likely that the actin filament network is anchored to the plasma membrane and matrix through a focal adhesion-like protein complex.

Actin dynamics are critical for the formation and/or maintenance of the sheath actin network. Actin depolymerizing factor (ADF)/cofilins promote rapid filament disassembly through monomer sequestration, filament severing and association with plus ends of filaments, which can prevent capping and accelerate plus-end depolymerization of the filaments [60]. In *C. elegans*, ADF/cofilin is encoded by *unc-60*, which is alternatively spliced to produce UNC-60A and UNC-60B [51,61,62]. UNC-60A, required for assembly of actin networks in the sheath, strongly binds G-actin monomers, but exhibits weak actin severing activity. The degree of actin severing activity is important, because UNC-60B cannot substitute for UNC-60A in the sheath unless the severing activity of UNC-60B is decreased by mutation [62]. In addition to regulation by alternative splicing, local alteration of ADF/cofilin activity by pH might influence the assembly and disassembly of the varied actin–filament networks seen in the somatic gonad [63]. Actin dynamics in the sheath are also regulated by two actin-interacting protein 1 (AIP1) homologs, UNC-78 and AIPL-1 [64]. AIP1 proteins enhance the capacity of cofilin to depolymerize actin [65]. Similarly to loss of cofilin, depletion of UNC-78 and AIPL-1 results in abnormal aggregation of actin in both the sheath and the spermatheca [66] (discussed below). These data suggest turnover of actin is important in both the sheath and spermatheca to maintain the characteristic actin structures.

## 3. The Spermatheca

The spermatheca, the morphogenesis of which occurs during L4 [40], consists of a single layer of 24 myoepithelial cells, is the site of sperm storage and fertilization [40,43]. Sheath cell contractions propel the oocyte into the spermatheca, dramatically stretching the tissue and initiating a process that culminates in spermathecal cell contraction and expulsion of the fertilized egg into the uterus [43]. Contraction of the spermatheca is thought to be driven by a mechanism similar to that observed in smooth muscle and non-muscle cells [67,68], where Ca^2+^–calmodulin activation of MLCK-1/myosin light chain kinase (MLCK) and RHO-1/Rho activation of LET-502/Rho kinase (ROCK) lead to the phosphorylation and activation of MLC-4/non-muscle myosin II (NM II) and contraction of acto-myosin fibers [52,69,70,71,72,73]. In addition, ROCK may also indirectly result in an increase in phospho-myosin levels through the inhibition of the MEL-11/the regulatory subunit of the myosin phosphatase [74]. Unlike the sheath, the spermatheca does not express the muscle-specific myosin heavy chains or the troponin complex [51].

The ovulation cycle, including oocyte entry, transit through the spermatheca, and expulsion into the uterus, repeats approximately every 20 min until roughly 150 eggs have been produced by each gonad arm [43]. Because the nematode is transparent, and the cells of the spermatheca clearly visible, the entire ovulatory process can be visualized using time-lapse microscopy. Each cell in the spermatheca has an invariant position and orientation [41], with bundles aligned parallel to the long axis of each cell (see Figure 1) [41,46,75,76].

The actin cytoskeleton plays a central role in spermathecal contractility [70,75,76,77,78]. The most prominent spermathecal actin cytoskeletal features are the stress-fiber-like acto-myosin bundles, which are oriented along the long axis of each cell [75]. Time lapse imaging revealed that these bundles develop concomitantly with contraction during the first ovulation and are maintained throughout subsequent ovulations [75]. Before ovulation, the actin cytoskeleton in the spermathecal cells is a poorly aligned, mesh-like network of thin fibers [75]. Oocyte entry leads to myosin activation and rapid maturation of the actin cytoskeleton into parallel, aligned acto-myosin bundles [75]. Manipulating the activation state of myosin genetically, for example through depletion of the MEL-11/myosin phosphatase, indicates that the degree of bundle formation and alignment is proportional to levels of myosin activity, with hyperactivation of myosin leading to the formation of large laterally associated conglomerations of actin fibers [75]. These results suggest that myosin can be activated by the stretch of oocyte entry, and that activated myosin then drives actin alignment and network organization in the *C. elegans* spermatheca.

How does the spermatheca ‘know’ that it has been stretched and how does stretch trigger contractility in this system? One of the mechanosensors is the Rho-GTPase activating protein (GAP) SPV-1. SPV-1 has an F-BAR domain that is predicted to fold into a banana-like shape, permitting SPV-1 to bind to curved or low-tension membranes. While at the membrane, SPV-1 acts as a GTPase activating protein GAP for RHO-1 and CDC-42, keeping both in the GDP-bound, inactive state. Oocyte entry into the spermatheca stretches the membrane and displaces SPV-1, allowing activation of RHO-1 and CDC-42. GTP-bound RHO-1 activates LET-502/ROCK, which activates contractility, and GTP-bound CDC-42 elevates calcium signaling through an undetermined signaling pathway [73,79]. Identifying other GTPases and upstream regulators such as guanine nucleotide dissociation (GDI) factors and guanine exchange factors (GEF) will be important for understanding how stretch triggers contractility in this system.

Tissue-level distribution of kinases can regulate the establishment of actin-based structures and acto-myosin contractility. In the spermatheca, both MLCK-1 and LET-502/ROCK are required for the maintenance of proper acto-myosin bundles and dynamics [70,75]. The two kinases stimulate acto-myosin contractility in subsets of spermathecal cells. ROCK/LET-502 is expressed primarily in the distal neck of the spermatheca and sp–ut valve [70,72,80], whereas MLCK-1 is highly expressed in the central cells of the spermathecal bag with some expression in the sp–ut valve [70]. Together, these two kinases coordinate ovulation in the *C. elegans* spermatheca, with MLCK stimulating the strong distal contractions that force the oocyte out into the spermatheca and ROCK controlling the distal neck closure and sp–ut valve contractility.

Once formed, the contractile acto-myosin bundles are maintained throughout adulthood, robust enough to withstand the transits of over one hundred embyros. Although the molecular mechanisms allowing for bundle maintenance during successive rounds of ovulation are largely unknown, several mechanisms have been identified. Filamin, known primarily for its actin cross-linking function, is a structural and signaling scaffold that binds transmembrane receptors and a wide variety of intracellular signaling proteins [81,82]. FLN-1/filamin is required to maintain the actin cytoskeleton in the spermatheca. In filamin-deficient animals, the spermatheca develops normally, and prior to the first ovulation, spermathecal F-actin is normal. However, F-actin bundles are not maintained and instead, actin progressively accumulates at cell-cell junctions [76]. In *fln-1* mutants, embryos are trapped in the spermatheca for extended periods of time and the spermatheca hyperextends to accommodate multiple ovulation events. With this excess stretch, the actin phenotype worsens suggesting FLN-1/filamin is necessary for maintenance of the aligned, evenly distributed, circumferential actin bundles under stress.

The *C. elegans* spectrins SPC-1/α spectrin, UNC-70/β spectrin, and SMA-1/β-heavy (βH) spectrin [83,84,85,86], also regulate acto-myosin network organization and robustness to mechanical stress in the spermatheca [87]. Spectrins, first discovered in erythrocytes, form a cortical network that in coordination with actin provides mechanical durability to cells [88,89,90]. The spectrin cytoskeleton also plays an important role in maintaining the tubular structure and robustness of neuronal axons [91]. Instead of forming thick, aligned bundles during contraction, actin bundles in cells lacking spectrin become thin and less well organized [87]. Depletion of SPC-1/α spectrin in adults using auxin initiated decay (AID) demonstrated that spectrin is needed for maintenance and robustness of the actin cytoskeleton to multiple rounds of stretch and contraction post-development [87]. These observations suggest that spectrin and the actin cytoskeleton likely work together to produce robust, flexible actin networks in contractile cells.

## 4. The SP–UT Valve

The spermatheca–uterine (sp–ut) valve is a donut-shaped syncytium of four cells (designated as sujn and sujc) that connects the spermatheca and the uterus [40]. Initially, two syncytial core cells (sujc) plug the core of the valve, but these are pushed out during the first ovulation [40]. SPC-1/α spectrin and SMA-1/βH spectrin co-localize at the juncture between the spermatheca and the sp–ut valve, suggesting a possible role in strengthening the attachment between these two tissues [87]. The most obvious actin structures in the sp–ut valve are the dense circumferential actin bundles that wrap around the core of the valve closer to the apical surface [41,75,76]. The valve contracts to prevent premature exit of the embryo into the uterus. When an oocyte enters the spermatheca and is fertilized, the valve remains closed during the period of eggshell formation. Premature expulsion of the fertilized egg can lead to egg shape defects and, in extreme cases, pinching off of pieces of cytoplasm if the valve closes on the unprotected embryo [73,76]. During embryo exit, the valve is pulled open by actin contraction in the proximal spermathecal cells, and the embryo is pushed into the uterus by contraction of the distal spermatheca. After embryo exit, the valve immediately recloses [70]. The actin cytoskeleton almost certainly plays an important role in the expansion and contraction of the sp–ut, as do the septate junctions (discussed below) which permit expansion of the valve syncytium [41]. However, very little is known about how the actin structures of the valve form and what mechanisms allow repeated dilation and contraction of the valve.

Contraction of the sp–ut valve requires myosin activity [70,75] and seems to be regulated primarily through Rho/ROCK [70]. Not only is LET-502/ROCK expressed at relatively higher levels in the valve compared to the central cells of the spermathecal bag [70,72,80], but loss of *let-502* results in a flaccid valve where embryos are released into the uterus prior to the beginning stages of eggshell formation [70,92]. When myosin activity is depleted by *nmy-1* RNAi, the usually tightly coiled core of the valve becomes large and loose, with individual actin bundles becoming visible [75]. These data suggest that myosin activity not only drives sp–ut constriction, regulating timing of ovulation, but also that phosphorylated myosin helps drive the formation of the tight, circumferential actin bundles of the valve. Similar ROCK-dependent tonic contraction has been seen in the rat internal anal sphincter (IAS), where inhibition of ROCK resulted in redistribution of ROCK and actin, and resulted in reduced tonic contractility [93].

Given that actin forms a dense actin network in the sp–ut valve, it should be unsurprising that actin crosslinkers are critical for both the actin structure and function of the valve. One actin crosslinker, FLN-1/filamin A, is expressed in both the spermatheca bag and sp–ut valve and is required to maintain proper valve morphology and actin structure [76,94]. When *fln-1* is depleted, the valve loses its typical toroidal shape and adopts a more crumpled appearance along the apical surface. In addition, the sp–ut valve does not contract in *fln-1* mutants, but rather it stays in a loose, open conformation. The actin structure appears stringy and disorganized, with most of the actin being pulled to the most distal and proximal edges of the valve [76]. In addition to filamin, another actin crosslinker, spectrin, is required for the integrity of the sp–ut valve during ovulations. In addition to being expressed in the spermatheca, the spectrin isoforms SPC-1/α spectrin, UNC-70/β spectrin, and SMA-1/βH spectrin all appear to be expressed in or around the sp–ut valve during L4 and adulthood [87]. Loss of *spc-1*/α-spectrin results in the valve becoming torn and the actin bundles appear disconnected only after the mechanical stress of an ovulation [87]. Together, these data suggest that Rho/ROCK is required to activate myosin to drive contraction of the actin bundles, but they need to be properly crosslinked into bundles and anchored to the spectrin network at the cell cortex for productive contractility and robustness to multiple ovulations.

## 5. Shared Features, Unanswered Questions, and Future Directions

Many unanswered questions remain regarding how actin alignment is established and maintained in *C. elegans*, and how similar cells establish strikingly different actin structures. For example, what mechanisms support the polymerization of the actin filaments that comprise the bundles? In many cell types, Arp2/3 and formins support the nucleation and elongation of actin filaments. For example, in *C. elegans* embryos, the Arp2/3 complex subunits (ARX-1, ARX-2, ARX-4, ARX-5, ARX-6 or ARX-7) and WSP-1/N-WASP are required for actin filament formation during ventral enclosure [95], and the formin FHOD-1 is required for actin filament formation in embryonic epidermal cells [96]. However, surprisingly little is known about the role of actin polymerization in the sheath or spermatheca. In the gonad, the formins FHOD-1 and EXC-6 are important for ovulation, but the spermathecal actin bundles are largely normal, even in the double mutant [78]. These results suggest redundant mechanisms may regulate the polymerization of actin filaments in the somatic gonad, and is an important area for future investigation.

Anchorage is required for bundles to withstand multiple rounds of stretch and contraction, and to convert contraction of the cytoskeleton to cell- or tissue-level contraction. In most contractile cell types, anchorage of the actin cytoskeleton occurs through integrin-based cell-matrix adhesions and/or through cadherin–catenin-based cell–cell junctions. As discussed above, the sheath cells contain dense bodies, structures analogous to focal adhesions that in mammalian cells anchor the acto-myosin rich stress fibers. Integrins are expressed in the spermatheca [97], where they may play a role in anchorage of the actin bundles, although the formation or role of focal adhesions in the spermatheca has not been studied.

Apical junctions, referred to as *C. elegans* apical junctions (CeAJ), help polarize *C. elegans* epithelial cells, provide barrier function, and attach to the cytoskeleton to provide transmission of mechanical information across tissues [98]. The CeAJ consists of an apical subdomain containing the PAR/aPKC complex which is crucial for establishing apical–basal polarity in epithelial tissues [80,98,99]. Slightly basal to that is the cadherin–catenin complex (CCC), a region containing HMR-1/E-cadherin, HMP-1/α-catenin, and HMP-2/β-catenin [98,100]. The CCC is required for ventral enclosure of the embryo, and directly binds and anchors actin [100]. Loss of either HMP-1/α-catenin or HMP-2/β-catenin in the embryo results in actin bundles becoming detached from the CeAJ [100]. The most basal structure is a complex made up of DLG-1/Discs large and AJM-1 and overlaps with the septate junction (discussed below) [98]. Loss of the PAR protein PAR-3 in the spermatheca results in the mislocalization of other CeAJ proteins, AJM-1 and LET-413/Scribble and consequent disruption of the actin bundle alignment and orientation [80,101]. The roles of the other CeAJ proteins in actin anchorage, and in the intercellular transmission of mechanical signals, have not been studied in the somatic gonad. Because CeAJ proteins are required for embryonic and larval development, the answers to these questions will require fine spatio-temporal control, perhaps using targeted auxin-initiated decay [102] to remove the potential anchors in a short time window in the specific cell type of interest.

Another likely relevant, but largely unstudied, structure found in the spermatheca and sp–ut valve are the septate junctions. Septate junctions (SJ) are tight junction-like structures found in invertebrates and are responsible for cell–cell adhesion and barrier function [103,104,105]. Pleated SJ are found apical to the CCC and fold the membrane into tight convolutions reminiscent of a piece of corrugated cardboard [41,106]. This feature allows septate junctions to be extended, or stretched out, without loss of barrier function as cells grow or stretch. For example, in Drosophila, the sub-perineural glia cells, which form the blood brain barrier, attach to one another via septate junctions. As these cells grow rapidly, the septate junctions allow for rapid expansion, going from wavy to straight as the cells elongate without any additional junction assembly [104,107]. Interestingly, actin-rich structures adjacent to these junctions are required for the continuity of the SJs [105]. Smooth or continuous SJ comprise long, continuous arrays of highly stable cell adhesion and other molecules [98,103,108,109]. In the *C. elegans* spermatheca, SJ-like structures are observed basally to the CeAJ [41,98], and probably play an important role in facilitating passage of embryos into and out of the spermatheca [101]. Similarly, when the sp–ut valve is closed, the apical surfaces of the inner part of the “donut” are convoluted and come in contact with one another. During embryo exit, these contacts are pulled apart and stretched considerably, but appear to reform immediately after closing (our unpublished observations). SJ proteins such as DLG-1/Discs large [101], and the neurexin homologs NRX-1 and ITX-1 are expressed in the sp–ut and spermatheca, respectively [110,111], and likely constitute core SJ components in the *C. elegans* gonad. It will be important to determine how these structures contribute to spermatheca and sp–ut valve distensibility and coordination of acto-myosin based contractility.

What is the relationship between cell shape and actin bundle orientation? In the spermatheca, actin bundles are aligned circumferentially around the spermathecal tube, which facilitates contraction of this tissue and expulsion of the fertilized egg [75]. Similarly, during embryonic elongation, actin bundles are arranged circumferentially in the epidermal cells, positioned to squeeze and elongate the round embryo into a worm shape. In contrast to the spermathecal actin bundles, which are arranged parallel to the long axis of the cells, actin in the embryonic epidermis is arranged perpendicularly to the long axis of the cell [7,100,112,113]. How do the fibers “know” to align in this stereotypical manner? Possibilities include polarized localization of actin nucleation, polymerization, severing or anchoring proteins, and/or a bias conferred by the tissue shape, the alignment of the extracellular matrix, or mechanically by the direction of strain applied to the actin filaments. Future imaging and modeling experiments will help to address these questions.

Experiments in which uniaxial cyclic strain has been applied to endothelial cells, fibroblasts and smooth muscle cells grown on flexible substrates suggest stress fibers align in the direction perpendicular to the direction of stretch [114]. This phenomenon is known as stretch (or strain) avoidance and is thought to be the result of destabilization and higher turnover of stress fibers under tension [114]. Despite this general principle, the exact response of the cytoskeleton to the mechanical perturbation is dependent on the specific experimental condition, focal adhesion attachment, matrix density cell type, and activation state of Rho [6,115,116]. It is not known whether stretch avoidance plays a role in actin fiber bundle orientation in *C. elegans*. In vivo tension measurements, perhaps using Fluorescence Resonance Energy Transfer (FRET)-based tension sensors [117] or laser severing experiments [118] may help answer the question of how actin bundles in *C. elegans* orient with respect to strain.

Much more than just a gene discovery platform, *C. elegans* provides a window into cell biology in the context of a living organism. Given the conservation of the genes encoding actin and actin regulatory proteins in *C. elegans*, and the significant advances in genome editing, in vivo imaging, ‘the worm’ will continue to enable discovery of the mechanism(s) by which the actin cytoskeleton is regulated at the intra- and inter-cellular levels.

## References

[1] Pollard T.D. (2016). Actin and Actin-Binding Proteins. Cold Spring Harb. Perspect. Biol..

[2] Plastino J., Blanchoin L. (2018). Dynamic stability of the actin ecosystem. J. Cell Sci..

[3] Velarde N., Gunsalus K.C., Piano F. (2007). Diverse roles of actin in *C. elegans* early embryogenesis. BMC Dev. Biol..

[4] Reymann A.-C., Staniscia F., Erzberger A., Salbreux G., Grill S.W. (2016). Cortical flow aligns actin filaments to form a furrow. Elife.

[5] Miller A.L. (2011). The contractile ring. Curr. Biol..

[6] Mueller J., Szep G., Nemethova M., de Vries I., Lieber A.D., Winkler C., Kruse K., Small J.V., Schmeiser C., Keren K. (2017). Load Adaptation of Lamellipodial Actin Networks. Cell.

[7] Williams-Masson E.M., Malik A.N., Hardin J. (1997). An actin-mediated two-step mechanism is required for ventral enclosure of the *C. elegans* hypodermis. Development.

[8] Burridge K., Wittchen E.S. (2013). The tension mounts: Stress fibers as force-generating mechanotransducers. J. Cell Biol..

[9] Tojkander S., Gateva G., Lappalainen P. (2012). Actin stress fibers—Assembly, dynamics and biological roles. J. Cell Sci..

[10] Blanchoin L., Boujemaa-Paterski R., Sykes C., Plastino J. (2014). Actin dynamics, architecture, and mechanics in cell motility. Physiol. Rev..

[11] Li S., Huang N.F., Hsu S. (2005). Mechanotransduction in endothelial cell migration. J. Cell. Biochem..

[12] Wozniak M.A., Chen C.S. (2009). Mechanotransduction in development: A growing role for contractility. Nat. Rev. Mol. Cell Biol..

[13] Nelson C.M., Gleghorn J.P. (2012). Sculpting Organs: Mechanical Regulation of Tissue Development. Annu. Rev. Biomed. Eng..

[14] Jodoin J.N., Coravos J.S., Perkins L.A., Perrimon N., Correspondence A.C.M., Chanet S., Vasquez C.G., Tworoger M., Kingston E.R., Martin A.C. (2015). Stable Force Balance between Epithelial Cells Arises from F-Actin Turnover Article. Dev. Cell.

[15] Brugues A., Anon E., Conte V., Veldhuis J.H., Gupta M., Colombelli J., Munoz J.J., Brodland G.W., Ladoux B., Trepat X. (2014). Forces driving epithelial wound healing. Nat. Phys..

[16] Davis M.J., Wu X., Nurkiewicz T.R., Kawasaki J., Davis G.E., Hill M.A., Meininger G.A. (2001). Integrins and mechanotransduction of the vascular myogenic response. Am. J. Physiol. Heart Circ. Physiol..

[17] Boudou T., Legant W.R., Mu A., Borochin M.A., Thavandiran N., Radisic M., Zandstra P.W., Epstein J.A., Margulies K.B., Chen C.S. (2012). A microfabricated platform to measure and manipulate the mechanics of engineered cardiac microtissues. Tissue Eng. Part A.

[18] Skwarek-Maruszewska A., Hotulainen P., Mattila P.K., Lappalainen P. (2009). Contractility-dependent actin dynamics in cardiomyocyte sarcomeres. J. Cell Sci..

[19] Greiner A.M., Chen H., Spatz J.P., Kemkemer R. (2013). Cyclic Tensile Strain Controls Cell Shape and Directs Actin Stress Fiber Formation and Focal Adhesion Alignment in Spreading Cells. PLoS ONE.

[20] Hannezo E., Dong B., Recho P., Joanny J.-F., Hayashi S. (2015). Cortical instability drives periodic supracellular actin pattern formation in epithelial tubes. Proc. Natl. Acad. Sci. USA.

[21] Mason F.M., Tworoger M., Martin A.C. (2013). Apical domain polarization localizes actin-myosin activity to drive ratchet-like apical constriction. Nat. Cell Biol..

[22] Hosono C., Matsuda R., Adryan B., Samakovlis C. (2015). Transient junction anisotropies orient annular cell polarization in the Drosophila airway tubes. Nat. Cell Biol..

[23] Pellegrin S., Mellor H. (2007). Actin stress fibres. J. Cell Sci..

[24] Naumanen P., Lappalainen P., Hotulainen P. (2008). Mechanisms of actin stress fibre assembly. J. Microsc..

[25] Pollard T.D., Blanchoin L., Mullins R.D. (2000). Molecular mechanisms controlling actin filament dynamics in nonmuscle cells. Annu. Rev. Biophys. Biomol. Struct..

[26] Pollard T.D., Cooper J.A. (2009). Actin, a Central Player in Cell Shape and Movement. Science.

[27] Cetera M., Ramirez-San Juan G.R., Oakes P.W., Lewellyn L., Fairchild M.J., Tanentzapf G., Gardel M.L., Horne-Badovinac S. (2014). Epithelial rotation promotes the global alignment of contractile actin bundles during Drosophila egg chamber elongation. Nat. Commun..

[28] Kasza K.E., Farrell D.L., Zallen J.A. (2014). Spatiotemporal control of epithelial remodeling by regulated myosin phosphorylation. Proc. Natl. Acad. Sci. USA.

[29] Higashi T., Arnold T.R., Stephenson R.E., Dinshaw K.M., Miller A.L. (2016). Maintenance of the Epithelial Barrier and Remodeling of Cell-Cell Junctions during Cytokinesis. Curr. Biol..

[30] Stephenson R.E., Higashi T., Erofeev I.S., Arnold T.R., Leda M., Goryachev A.B., Miller A.L. (2019). Rho Flares Repair Local Tight Junction Leaks. Dev. Cell.

[31] Mandato C.A., Bement W.M. (2001). Contraction and polymerization cooperate to assemble and close actomyosin rings around Xenopus oocyte wounds. J. Cell Biol..

[32] Merriam R.W., Christensen K. (1983). A contractile ring-like mechanism in wound healing and soluble factors affecting structural stability in the cortex of Xenopus eggs and oocytes. J. Embryol. Exp. Morphol..

[33] Sonnemann K.J., Bement W.M. (2011). Wound Repair: Toward Understanding and Integration of Single-Cell and Multicellular Wound Responses. Annu. Rev. Cell Dev. Biol..

[34] Sherwood D.R., Plastino J. (2018). Invading, leading and navigating cells in caenorhabditis elegans: Insights into cell movement in vivo. Genetics.

[35] Tang N.H., Jin Y. (2018). Shaping neurodevelopment: Distinct contributions of cytoskeletal proteins. Curr. Opin. Neurobiol..

[36] Ono S. (2014). Regulation of structure and function of sarcomeric actin filaments in striated muscle of the nematode *Caenorhabditis elegans*. Anat. Rec..

[37] Hubbard E.J.A., Greenstein D. (2000). The *Caenorhabditis elegans* Gonad: A Test Tube for Cell and Developmental Biology. Dev. Dyn..

[38] Whitten S.J., Miller M.A. (2006). The role of gap junctions in *Caenorhabditis elegans* oocyte maturation and fertilization. Dev. Biol..

[39] Altun Z.F., Chen B., Wang Z.-W., Hall D.H. (2009). High resolution map of *Caenorhabditis elegans* gap junction proteins. Dev. Dyn..

[40] Kimble J., Hirsh D. (1979). The postembryonic cell lineages of the hermaphrodite and male gonads in *Caenorhabditis elegans*. Dev. Biol..

[41] Lints R., Hall D.H. (2009). Reproductive system, somatic gonad. WormAtlas.

[42] McCarter J., Bartlett B., Dang T., Schedl T. (1997). Soma–Germ Cell Interactions in *Caenorhabditis elegans*: Multiple Events of Hermaphrodite Germline Development Require the Somatic Sheath and Spermathecal Lineages. Dev. Biol..

[43] McCarter J., Bartlett B., Dang T., Schedl T. (1999). On the Control of Oocyte Meiotic Maturation and Ovulation in *Caenorhabditis elegans*. Dev. Biol..

[44] Grant B., Hirsh D. (1999). Receptor-mediated Endocytosis in the *Caenorhabditis elegans* Oocyte. Mol. Biol. Cell.

[45] Hall D.H., Winfrey V.P., Blaeuer G., Hoffman L.H., Furuta T., Rose K.L., Hobert O., Greenstein D. (1999). Ultrastructural Features of the Adult Hermaphrodite Gonad of *Caenorhabditis elegans*: Relations between the Germ Line and Soma. Dev. Biol..

[46] Strome S. (1986). Fluorescence Visualization of the Distribution of Microfilaments in Gonads and Early Embryos of the Nematode *Caenorhabditis elegans*. J. Cell Biol..

[47] Yin X.Y., Gower N.J.D., Baylis H.A., Strange K. (2004). Inositol 1,4,5-Trisphosphate Signaling Regulates Rhythmic Contractile Activity of Myoepithelial Sheath Cells in *Caenorhabditis elegans*. Mol. Biol. Cell.

[48] Clandinin T.R., DeModena J.A., Sternberg P.W. (1998). Inositol trisphosphate mediates a RAS-independent response to LET-23 receptor tyrosine kinase activation in *C. elegans*. Cell.

[49] Norman K.R., Fazzio R.T., Mellem J.E., Espelt M.V., Strange K., Beckerle M.C., Maricq A.V. (2005). The Rho/Rac-Family Guanine Nucleotide Exchange Factor VAV-1 Regulates Rhythmic Behaviors in *C. elegans*. Cell.

[50] Ardizzi J.P., Epstein H.F. (1987). Immunochemical Localization of Myosin Heavy Chain Isoforms and Paramyosin in Developmentally and Structurally Diverse Muscle Cell Types of the Nematode *Caenorhabditis elegans*. J. Cell Biol..

[51] Ono K., Yu R., Ono S. (2007). Structural components of the nonstriated contractile apparatuses in the *Caenorhabditis elegans* gonadal myoepithelial sheath and their essential roles for ovulation. Dev. Dyn..

[52] Ono K., Ono S. (2016). Two distinct myosin II populations coordinate ovulatory contraction of the myoepithelial sheath in the *Caenorhabditis elegans* somatic gonad. Mol. Biol. Cell.

[53] Obinata T., Ono K., Ono S. (2010). Troponin I controls ovulatory contraction of non-striated actomyosin networks in the *C. elegans* somatic gonad. J. Cell Sci..

[54] Ono K., Ono S. (2004). Tropomyosin and Troponin Are Required for Ovarian Contraction in the *Caenorhabditis elegans* Reproductive System. Mol. Biol. Cell.

[55] Myers C.D., Goh P.Y., Allen T.S., Bucher E.A., Bogaert T. (1996). Developmental genetic analysis of troponin T mutations in striated and nonstriated muscle cells of *Caenorhabditis elegans*. J. Cell Biol..

[56] Geiger B., Spatz J.P., Bershadsky A.D. (2009). Environmental sensing through focal adhesions. Nat. Rev. Mol. Cell Biol..

[57] Ciobanasu C., Faivre B., Le Clainche C. (2012). Actin Dynamics Associated with Focal Adhesions. Int. J. Cell Biol..

[58] Xu X., Rongali S.C., Miles J.P., Lee K.D., Lee M. (2006). pat-4/ILK and unc-112/Mig-2 are required for gonad function in *Caenorhabditis elegans*. Exp. Cell Res..

[59] Cram E.J., Clark S.G., Schwarzbauer J.E. (2003). Talin loss-of-function uncovers roles in cell contractility and migration in *C. elegans*. J. Cell Sci..

[60] Wioland H., Guichard B., Senju Y., Myram S., Lappalainen P., Jégou A., Romet-Lemonne G. (2017). ADF/Cofilin Accerlerates Actin Dynamics by Severing Filaments and Promoting Their Depolymerization at Both Ends. Curr. Biol..

[61] Ono S., Baillie D.L., Benian G.M. (1999). UNC-60B, an ADF/Cofilin Family Protein, Is Required for Proper Assembly of Actin into Myofibrils in *Caenorhabditis elegans* Body Wall Muscle. J. Cell Biol..

[62] Ono K., Yamashiro S., Ono S. (2008). Essential role of ADF/cofilin for assembly of contractile actin networks in the *C. elegans* somatic gonad. J. Cell Sci..

[63] Nomura K., Hayakawa K., Tatsumi H., Ono S. (2016). Actin-interacting Protein 1 Promotes Disassembly of Actin-depolymerizing Factor/Cofilin-bound Actin Filaments in a pH-dependent Manner. J. Biol. Chem..

[64] Ono K., Ono S. (2014). Two actin-interacting protein 1 isoforms function redundantly in the somatic gonad and are essential for reproduction in *Caenorhabditis elegans*. Cytoskeleton.

[65] Rodal A.A., Tetreault J.W., Lappalainen P., Drubin D.G., Amberg D.C. (1999). Aip1p Interacts with Cofilin to Disassemble Actin Filaments. J. Cell Biol..

[66] Ono S. (2018). Functions of actin-interacting protein 1 (AIP1)/WD repeat protein 1 (WDR1) in actin filament dynamics and cytoskeletal regulation. Biochem. Biophys. Res. Commun..

[67] Zaidel-Bar R., Joyce M.J., Lynch A.M., Witte K., Audhya A., Hardin J. (2010). The F-BAR domain of SRGP-1 facilitates cell-cell adhesion during *C. elegans* morphogenesis. J. Cell Biol..

[68] Hong F., Haldeman B.D., Jackson D., Carter M., Baker J.E., Cremo C.R. (2011). Biochemistry of Smooth Muscle Myosin Light Chain Kinase. Arch. Biochem. Biophys..

[69] Kovacevic I., Orozco J.M., Cram E.J. (2013). Filamin and phospholipase C-ε are required for calcium signaling in the *Caenorhabditis elegans* spermatheca. PLoS Genet..

[70] Kelley C.A., Wirshing A.C.E., Zaidel-Bar R., Cram E.J. (2018). The myosin light-chain kinase MLCK-1 relocalizes during *Caenorhabditis elegans* ovulation to promote actomyosin bundle assembly and drive contraction. Mol. Biol. Cell.

[71] Wissmann A., Ingles J., McGhee J.D., Mains P.E. (1997). *Caenorhabditis elegans* LET-502 is related to Rho-binding kinases and human myotonic dystrophy kinase and interacts genetically with a homolog of the regulatory subunit of smooth muscle myosin phosphatase to affect cell shape. Genes Dev..

[72] Wissmann A., Ingles J., Mains P.E. (1999). The *Caenorhabditis elegans* mel-11 Myosin Phosphatase Regulatory Subunit Affects Tissue Contraction in the Somatic Gonad and the Embryonic Epidermis and Genetically Interacts with the Rac Signaling Pathway. Dev. Biol..

[73] Tan P.Y., Zaidel-Bar R. (2015). Transient membrane localization of SPV-1 drives cyclical actomyosin contractions in the *C. elegans* spermatheca. Curr. Biol..

[74] Kimura K., Ito M., Amano M., Chihara K., Fukata Y., Nakafuku M., Yamamori B., Feng J., Nakano T., Okawa K. (1996). Regulation of Myosin Phosphatase by Rho and Rho-Associated Kinase (Rho-Kinase). Science.

[75] Wirshing A.C.E., Cram E.J. (2017). Myosin activity drives actomyosin bundle formation and organization in contractile cells of the *Caenorhabditis elegans* spermatheca. Mol. Biol. Cell.

[76] Kovacevic I., Cram E.J. (2010). FLN-1/Filamin is required for maintenance of actin and exit of fertilized oocytes from the spermatheca in *C. elegans*. Dev. Biol..

[77] Deng H., Xia D., Fang B., Zhang H. (2007). The flightless I homolog, fli-1, regulates anterior/posterior polarity, asymmetric cell division and ovulation during *Caenorhabditis elegans* development. Genetics.

[78] Hegsted A., Wright F.A., Votra S., Pruyne D. (2016). INF2- and FHOD-related formins promote ovulation in the somatic gonad of *C. elegans*. Cytoskeleton.

[79] Bouffard J., Cecchetelli A.D., Clifford C., Sethi K., Zaidel-Bar R., Cram E.J. (2019). The RhoGAP SPV-1 regulates calcium signaling to control the contractility of the *C. elegans* spermatheca during embryo transits. Mol. Biol. Cell.

[80] Aono S., Legouis R., Hoose W.A., Kemphues K.J. (2004). PAR-3 is required for epithelial cell polarity in the distal spermatheca of *C. elegans*. Development.

[81] Kim H., McCulloch C.A. (2011). Filamin A mediates interactions between cytoskeletal proteins that control cell adhesion. FEBS Lett..

[82] Djinovic-Carugo K., Carugo O. (2010). Structural portrait of filamin interaction mechanisms. Curr. Protein Peptide Sci..

[83] McKeown C., Praitis V., Austin J. (1998). sma-1 encodes a betaH-spectrin homolog required for *Caenorhabditis elegans* morphogenesis. Development.

[84] Buechner M., Hall D.H., Bhatt H., Hedgecock E.M. (1999). Cystic canal mutants in *Caenorhabditis elegans* are defective in the apical membrane domain of the renal (excretory) cell. Dev. Biol..

[85] Norman K.R., Moerman D.G. (2002). α spectrin is essential for morphogenesis and body wall muscle formation in *Caenorhabditis elegans*. J. Cell Biol..

[86] Ferrier A., Charron A., Sadozai Y., Switaj L., Szutenbach A., Smith P.A. (2011). Multiple phenotypes resulting from a mutagenesis screen for pharynx muscle mutations in *Caenorhabditis elegans*. PLoS ONE.

[87] Wirshing A.C.E., Cram E.J. (2018). Spectrin regulates cell contractility through production and maintenance of actin bundles in the *Caenorhabditis elegans* spermatheca. Mol. Biol. Cell.

[88] Yu J., Fischman D.A., Steck T.L. (1973). Selective solubilization of proteins and phospholipids from red blood cell membranes by nonionic detergents. J. Supramol. Struct..

[89] Greenquist A.C., Shohet S.B., Bernstein S.E. (1978). Marked reduction of spectrinin hereditary spherocytosis in the common house mouse. Blood.

[90] Tse W.T., Lecomte M.C., Costa F.F., Garbarz M., Feo C., Boivin P., Dhermy D., Forget B.G. (1990). Point mutation in the beta-spectrin gene associated with alpha I/74 hereditary elliptocytosis. Implications for the mechanism of spectrin dimer self-association. J. Clin. Investig..

[91] Hammarlund M., Jorgensen E.M., Bastiani M.J. (2007). Axons break in animals lacking β-spectrin. J. Cell Biol..

[92] Maruyama R., Velarde N.V., Klancer R., Gordon S., Kadandale P., Parry J.M., Hang J.S., Rubin J., Stewart-Michaelis A., Schweinsberg P. (2007). EGG-3 Regulates Cell-Surface and Cortex Rearrangements during Egg Activation in *Caenorhabditis elegans*. Curr. Biol..

[93] Rattan S., De Godoy M.A.F., Patel C.A. (2006). Rho Kinase as a Novel Molecular Therapeutic Target for Hypertensive Internal Anal Sphincter. Gastroenterology.

[94] DeMaso C.R., Kovacevic I., Uzun A., Cram E.J. (2011). Structural and functional evaluation of *C. elegans* filamins FLN-1 and FLN-2. PLoS ONE.

[95] Sawa M., Suetsugu S., Sugimoto A., Miki H., Yamamoto M., Takenawa T. (2003). Essential role of the *C. elegans* Arp2/3 complex in cell migration during ventral enclosure. J. Cell Sci..

[96] Vanneste C.A., Pruyne D., Mains P.E. (2013). The role of the formin gene fhod-1 in *C. elegans* embryonic morphogenesis. Worm.

[97] Gettner S.N., Kenyon C., Reichardt L.E. (1995). Characterization of/3pat-3 Heterodimers, a Family of Essential Integrin Receptors in *C. elegans*. J. Cell Biol..

[98] Lynch A.M., Hardin J. (2009). The assembly and maintenance of epithelial junctions in *C. elegans*. Front. Biosci..

[99] Nance J., Munro E.M., Priess J.R. (2003). *C. elegans* PAR-3 and PAR-6 are required for apicobasal asymmetries associated with cell adhesion and gastrulation. Development.

[100] Costa M., Raich W., Agbunag C., Leung B., Hardin J., Priess J.R. (1998). A Putative Catenin-Cadherin System Mediates Morphogenesis of the *Caenorhabditis elegans* Embryo. J. Cell Biol..

[101] Pilipiuk J., Lefebvre C., Wiesenfahrt T., Legouis R., Bossinger O. (2009). Increased IP_3_/Ca^2+^ signaling compensates depletion of LET-413/DLG-1 in *C. elegans* epithelial junction assembly. Dev. Biol..

[102] Zhang L., Ward J.D., Cheng Z., Dernburg A.F. (2015). The auxin-inducible degradation (AID) system enables versatile conditional protein depletion in *C. elegans*. Development.

[103] Izumi Y., Furuse M. (2014). Molecular organization and function of invertebrate occluding junctions. Semin. Cell Dev. Biol..

[104] Babatz F., Naffin E., Klämbt C. (2018). The Drosophila Blood-Brain Barrier Adapts to Cell Growth by Unfolding of Pre-existing Septate Junctions. Dev. Cell.

[105] Hatan M., Shinder V., Schnorrer F., Volk T. (2011). The Drosophila blood brain barrier is maintained by GPCR-dependent dynamic actin structures. J. Cell Biol..

[106] Simske J.S. (2013). Claudins reign: The claudin/EMP/PMP22/γ channel protein family in *C. elegans*. Tissue Barriers.

[107] Oshima K., Fehon R.G. (2011). Analysis of protein dynamics within the septate junction reveals a highly stable core protein complex that does not include basolateral polarity protein Discs large. J. Cell Sci..

[108] Wiener J., Spiro D., Loewenstein W.R. (1964). Studies on an epithelial (gland) cell junction. II. Surfact Structure. J. Cell Biol..

[109] Noirot-Timothee C., Smith D.S., Cayer M.L., Noirot C. (1978). Septate Junctions in Insects: Comparison between Intercellular and Intramembranous Structures. Tissue Cell.

[110] Genova J.L., Fehon R.G. (2003). Neuroglian, Gliotactin, and the Na/K ATPase are essential for septate junction function in Drosophila. J. Cell Biol..

[111] Haklai-Topper L., Soutschek J., Sabanay H., Scheel J., Hobert O., Peles E. (2011). The neurexin superfamily of *Caenorhabditis elegans*. Gene Expr. Patterns.

[112] Costa M., Draper B.W., Priess J.R. (1997). The Role of Actin Filaments in Patterning the *Caenorhabditis elegans* Cuticle. Dev. Biol..

[113] Priess J.R., Hirsh D.I. (1986). *Caenorhabditis elegans* morphogenesis: The role of the cytoskeleton in elongation of the embryo. Dev. Biol..

[114] Tamiello C., Buskermolen A.B.C., Baaijens F.P.T., Broers J.L.V., Bouten C.V.C. (2015). Heading in the Right Direction: Understanding Cellular Orientation Responses to Complex Biophysical Environments. Cell. Mol. Bioeng..

[115] Plastino J., Blanchoin L. (2017). Adaptive Actin Networks. Dev. Cell.

[116] Kaunas R., Nguyen P., Usami S., Chien S. (2005). Cooperative effects of Rho and mechanical stretch on stress fiber organization. Proc. Natl. Acad. Sci. USA.

[117] LaCroix A.S., Rothenberg K.E., Berginski M.E., Urs A.N., Hoffman B.D. (2015). Construction, imaging, and analysis of FRET-based tension sensors in living cells. Methods Cell Biol..

[118] Kobb A.B., Zulueta-Coarasa T., Fernandez-Gonzalez R. (2017). Tension regulates myosin dynamics during *Drosophila* embryonic wound repair. J. Cell Sci..

